# Synthesis and characterization of new dialkylacylphosphonylhydrazones

**DOI:** 10.1016/j.dib.2018.08.073

**Published:** 2018-08-30

**Authors:** Henriqueta Talita Guimarães Barboza, Antonio Gomes Soares, Otniel Freitas-Silva, João Batista Neves DaCosta

**Affiliations:** a Embrapa Agroindústria de alimentos, Technology, Avenida das Américas, 29501, Guaratiba, CEP: 23020- 470, Rio de Janeiro, RJ, Brazil; b Federal Rural University of Rio de Janeiro (UFRRJ), BR-465, Km 7, Seropédica, CEP: 23.890-000, Rio de Janeiro, RJ, Brazil

## Abstract

The present work refers to the synthesis of novel dialkylacylphosphonylhydrazones that occurs in three reaction steps: the first one is the synthesis of different dialkyl acetate phosphonoacetates obtained by the reaction of ethyl bromoacetate with the trialkyl phosphite of interest. The second one is the synthesis of acetic diethoxyphosphonylhydrazines which is from the reaction between the synthesized dialkyl phosphonoacetates and hydrazine. The third and final steps is the condensation of acetic diethoxyphosphonylhydrazides with different heterocyclic aldehydes. In total, 17 unpublished compounds, namely 1 to 17 (Table 1) were obtained with a diastereoisomeric mixture of the preferential conformation *E* and all the compounds were characterized by 1-H and 13-C and 31-P NMR, infrared (IR) and mass spectroscopy (MS). This work presents the characterization data of these compounds.

**Specifications Table**

**Table:** 

Subject area	Chemistry
More specific subject area	Organic chemistry, organophosphorus
Type of data	Text file
How data was acquired	NMR (1H NMR (400 MHz), 13-C (100 MHz) and 31P (162 MHz) Bruker models Avance III 500 / Ultrashield 500 Plus and Avance II 400 / Ultrashield 400 Plus); mass spectroscopy (CG-MS - model QP2010 Plus - Shimadzu); infrared (FT-IR VERTEX 70).
Data format	Analyzed
Experimental factors	For the purification of the synthesized compounds, Biotagedo Isolera ™ Prime was used under the following analysis conditions: Ultra 10 g SNAP Cartridge - 25 μm silica stationary phase; wavelength detection mode; flow rate of 12 ml / min; Baseline correction, UV1 monitor readings at 254 nm and UV2 monitor readings at 365 nm and initial threshold of 20 mAU.
Experimental features	In the first step, in a 50 mL round bottom flask with a reflux condenser and bubbler, ethyl α-bromoacetate and triethyl or tributyl phosphite were reacted in slight excess. The reaction mixture remained under reflux and magnetic stirring for 6 h at 100 °C and was subsequently subjected to vacuum on the rotary evaporator for 5 h at 80 °C to remove excess of the remaining reagent. In the second step the triethyl phosphonoacetate, obtained above, was added to hydrazine monohydrate (64%) in a 50 mL round bottom flask, coupled to the rotary evaporator. The reaction mixture was kept under vacuum at 50 °C for 3 h. In the last step the diethoxyphosphonylhydrazides from step 2 was combined with the corresponding aldehyde, both previously dissolved in 3 mL of EtOH in a 50 mL round bottom flask. Then two 2 drops of 37% HCl were added. The reaction mixture was kept under stirring for 5 h at room temperature. After the reaction time had elapsed, the reaction medium was poured into ice-cold distilled water and left in an ice bath for half-an-hour for precipitation to occur. At the end of this time it was vacuum filtered and air dried. In cases where there was no precipitation, drops of 15% sodium bicarbonate solution were added to reach neutral pH. The resulting aqueous solution was treated with dichloromethane (4 ×15 mL). Finally, anhydrous Na_2_ SO_4_ was added to the organic solution, then it was filtered and evaporated in a rotary evaporator.
Data source location	–
Data accessibility	–

**Value of the data**

• The synthesis of these dialkylacylphosphonylhydrazones is interesting because of the potential biological activity of the clusters present in the structures as acylidrazones (—CO—NH—N=). The dialkylacylphosphonylhydrazones have applications ranging from medicinal compounds and agrochemicals up to functional materials and considering that organophosphates present effective agricultural protection.
• These compounds have potential drug action to combat Alzheimer׳s disease, where acetylcholinesterase inhibitor drugs are used. Initial studies have shown inhibition of the enzyme acetylcholinesterase, target of the drugs used for Alzheimer׳s disease. However, further studies should be conducted in order to verify their action without compromising human health.
• The steps to synthesize these compounds and their characterization are easily performed in the laboratory and there are a range of other products that can be obtained through this synthetic route.

## 1 Data

**Fig. 1:**
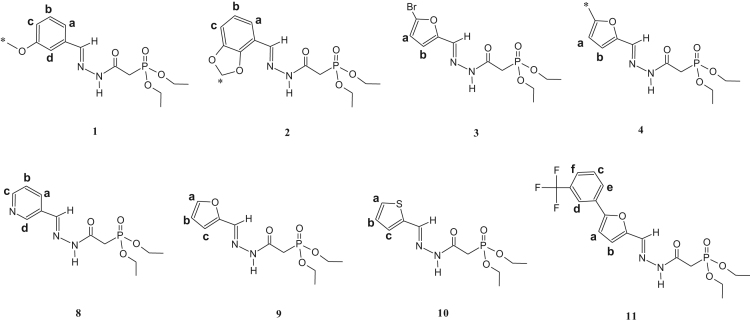
Compounds described in Table 2.

**Fig. 2:**
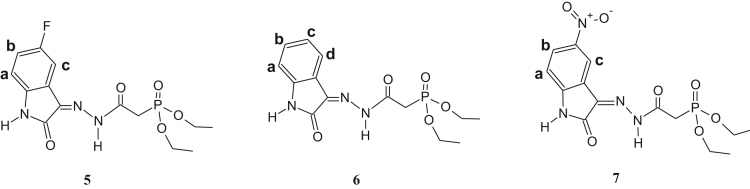
Compounds described in Table 3.

**Fig. 3:**
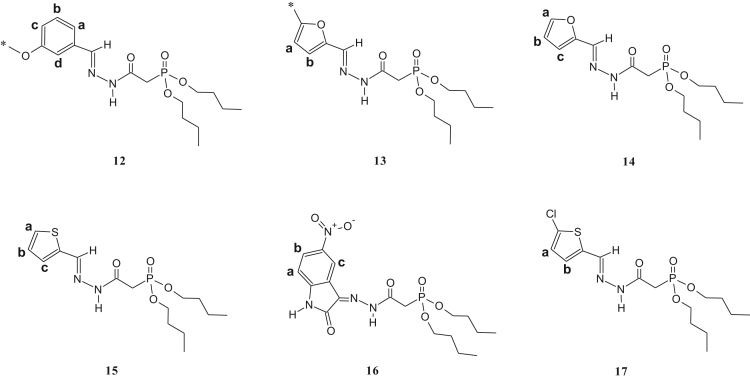
Compounds described in Table 4.

**Fig. 4:**
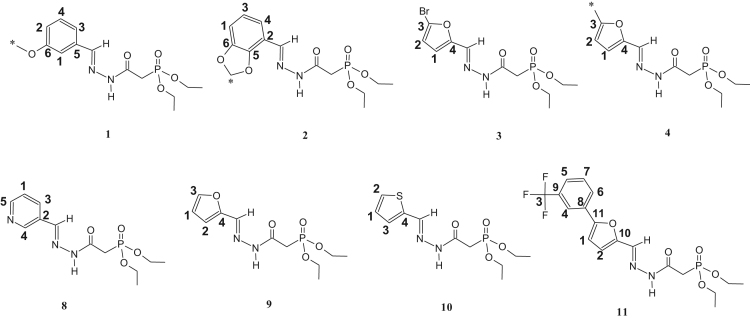
Compounds described in Table 5.

**Fig. 5:**
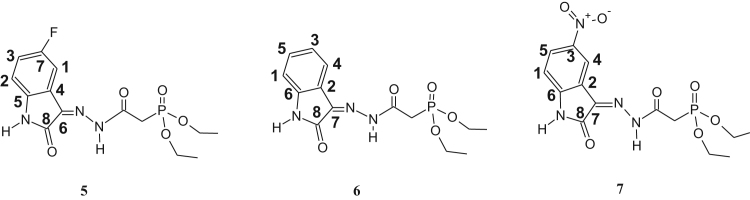
Compounds described in Table 6.

**Fig. 6:**
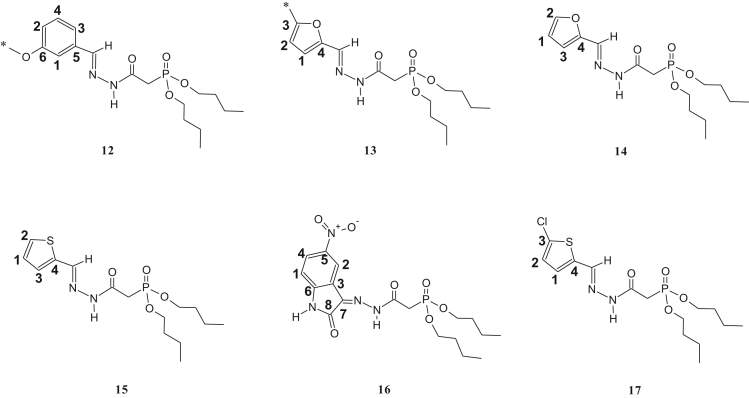
Compounds described in Table 7.

The synthesized dialkylphosphonylacylidrazones compounds are novel and were characterized by 1-H, 13-C and 31-P NMR as well as infrared (IR) and mass spectroscopy (MS). Due to the mixture of diastereoisomer obtained, duplications of the results in the presented spectra were observed. The ratio of the diastereoisomers is described in Table 9, which is preferred for the diastereoisomer E due to the prior works [1], [2], [3], [4], [5], [6], [7] (Fig. 1, Fig. 2, Fig. 3, Fig. 4, Fig. 5, Fig. 6).

**Table 1:** IR data of the compounds synthesized in cm^-1^.

* Insufficient sample to perform this analysis.

**Table 2:** 1-H NMR δ (ppm) data of the compounds synthesized from the reaction of intermediate III and aldehydes.

: Results obtained with DMSO as solvent, others results were obtained in CDCl_3_.

**Table 3:** 1-H NMR δ (ppm) data of the compounds synthesized from the reaction of intermediate III and isatins.

Results obtained with CDCl_3_ as solvent.

**Table 4:** 1-H NMR δ (ppm) data of the compounds synthesized from the reaction of intermediate IV with aldehydes and nitroisatin.

Results obtained with DMSO as solvent. •δ (ppm) relative to H of the isatin ring NHC=O, as shown in Table 6. Compound 16 is the only isatin derivative in this Table; all the others are aldehyde derivatives.

**Table 5:** 13- C NMR δ (ppm) data of the compounds synthesized from the reaction of intermediate III and aldehydes.

: Results obtained with CDCl_3_ as solvent. NA indicates that the said signal does not show in the spectrum.

**Table 6:** 13- C NMR δ (ppm) data of the compounds synthesized from the reaction of intermediate III and isatins.

*Results obtained with DMSO as solvent, the other results were obtained in CDCl_3_.

**Table 7:** 13-C NMR δ (ppm) data of the compounds synthesized from the reaction of intermediate IV and aldehydes.

Results obtained in DMSO as solvent. NA indicates that the said signal does not appear in the spectrum. Compound 16 is a compound derived from the reaction with nitroisatin.

**Table 8:** 31- P NMR δ (ppm) data of the compounds synthesized.

Compounds	31 P NMR (m/m) (coupled)	31 P NMR (s/s) (uncoupled)
1	21.20; 22.40	21.20; 22.40
2	21.23; 22.33	21.23; 22.33
3	20.98; 22.09	20.98; 22.09
4	21.24; 22.29	16.47; 17.56^a^
5	19.95; 20.10	19.94; 20.08
6	20.08; 20.19	20.04; 20.19
7	20.20; 20.58	20.20; 20.58
8	21.15; 22.09	21.13; 22.09
9	21.49; 21.80	21.49; 21.80
10	21.15; 22.35	21.16; 22.35
11	21.27; 22.12	21.26; 22.12
12	21.57; 21.92	21.57; 21.92
13^a^	21.63; 21.91	21.63; 21.91
14	21.48; 21.80	–
15^a^	21.49; 21.87	21.49; 21.87
16^a^	20.66; 20.17	20.66; 20.17
17^a^	20.51; 21.28	20.49; 21.26

a Results obtained with DMSO as solvent, others results were obtained in CDCl_3_.

**Table 9:** Ratio of the diastereoisomers in the mixture.

Compounds	Mix of diastereomeric	Compounds	Mix of diastereomeric
1	1:0.6	10	1:0.5
2	1:0.6	11	1:0.3
3	1:0.3	12	1:0.6
4	1: 0.5	13	1:0.75
5	1:0.4	14	1:0.7
6	1:0.4	15	1:0.9
7	1:0.6	16	1:0.4
8	1:0.8	17	1:0.4
9	1:0.7	–	–

**Table 10:** Mass data of the synthesized compounds.

Compounds	Molecular mass	m/z (%)
1	328	329 (3), 195 (14), 177 (50), 151 (64), 125 (92), 123 (100), 109 (52), 97 (46), 81 (31), 59 (35).
2	342	343 (4), 195 (32), 168 (50), 164 (50), 151 (77), 125 (100), 97 (50), 81 (38), 59 (39).
3	367	169 (35), 151 (33), 141 (15), 123 (100), 105 (29), 97 (64), 81 (49), 65 (35).
4	302	303 (5), 302 (22), 168 (22), 151 (31), 125 (38), 124 (100), 123 (45), 109 (37), 97 (20), 81 (26), 59 (13).
5	357	357 (5), 329 (18), 281 (11), 207 (17), 179 (37), 152 (86), 151 (40), 123 (100), 81 (55), 65 (17).
6	339	329 (18), 281 (9), 207 (14), 205 (17), 179 (37), 152 (85), 123 (100), 81 (53), 65 (17).
7	384	*
8	299	300 (1), 179 (<1), 152 (12), 120 (100), 109 (22), 92 (59), 65 (12).
9	288	289 (<1), 195 (13), 179 (14), 151 (45), 152 (25), 123 (68), 110 (100), 81 (36), 59 (14).
10	304	305 (4), 195 (30), 151 (77), 123 (100), 109 (72), 81 (47), 59 (31).
11	432	433 (1), 254 (100), 237 (53), 168 (42), 151 (57), 109 (37), 79 (19).
12	384	385 (1), 255 (13), 196 (14), 177 (33), 140 (88), 123 (100), 97 (86), 57 (21).
13	358	359 (6), 196 (11), 153 (14), 140 (72), 124 (100), 97 (52), 41 (23).
14	344	345 (2), 196 (14), 153 (17), 140 (70), 123 (94), 110 (100), 97 (55), 57 (21).
15	360	361 (2), 196 (23), 153 (24), 140 (91), 123 (100), 97 (77), 57 (23).
16	440	*
17	395	395 (1), 196 (20), 160 (38), 140 (84), 123 (100), 97 (62), 57 (22).

*There was no result in the mass spectrum.

## 2 Experimental design, materials and methods

Synthesis of dialkyl ethyl phosphonoacetate: triethyl phosphonoacetate (I) and dibutyl ethyl phosphonoacetate (II) (reaction intermediates to prepare intermediates III and IV) [5].

### 2.1 General procedure

In a 50 mL round bottom flask equipped with a reflux condenser provided with a bubbler, ethyl α-bromoacetate and triethyl or tributyl phosphite are added in a slight excess to ensure the total consumption of ethyl α-bromoacetate. The reaction mixture was kept under reflux and with magnetic stirring for 6 h at 100 ° C. At the end of the reaction the solution was subjected to vacuum on the rotary evaporator for 5 h at a temperature of 80 ° C in order to remove the excess of the remaining reagent.

Synthesis of dialkyl-2-hydrazino-2-oxyethyl: diethyl-2-hydrazino-2-oxyethyl phosphonate (III) and diethyl-2-hydrazino-2-oxyethyl phosphonate (IV) (reaction intermediates to prepare diethoxyphosphonyl-n-acylhydrazones [5] (Table 8, Table 10).

### 2.2 General procedure

The triethyl phosphonoacetate and the monohydrated hydrazine (64%) in a 50 mL round bottom flask are connected to a rotary evaporator. Then the reaction mixture is kept under vacuum at 50 ° C for 3 h.

Synthesis of diethoxyphosphonyl-n-acylhydrazones.

### 2.3 General procedure

The solubilized intermediates III or IV, and the corresponding aldehydes dissolved in 3 mL of EtOH were added to a 50 mL round bottom flask, followed by two 2 drops of 37% HCl. The reaction mixture was kept under stirring for 5 h at room temperature. After the reaction time had elapsed, the reaction medium was poured into ice-cold distilled water and left in an ice bath for half an hour for precipitation to occur. At the end of this time it was vacuum filtered and air dried. When there was no precipitation, drops of 15% sodium bicarbonate solution were added to reach neutral pH. The resulting aqueous solution was extracted with dichloromethane (4 ×15 mL). Then anhydrous Na_2_ SO_4_ was added to the organic solution, after which it was filtered and evaporated in a rotary evaporator.
